# Permanent tooth agenesis in non-syndromic Robin sequence and cleft palate: prevalence and patterns

**DOI:** 10.1007/s00784-016-2020-z

**Published:** 2016-12-09

**Authors:** Anneline de Smalen, Daan P. F. van Nunen, Ruurd R. Hermus, Edwin M. Ongkosuwito, Arjen J. van Wijk, J. Peter W. Don Griot, Corstiaan C. Breugem, Gem J. C. Kramer

**Affiliations:** 1 Department of Orthodontics, Academic Centre for Dentistry Amsterdam (ACTA), Gustav Mahlerlaan 3004, 1081 LA Amsterdam, The Netherlands; 2 Cleft Center Utrecht, Division of Plastic and Reconstructive Surgery, Wilhelmina Children’s Hospital, University Medical Center Utrecht, Heidelberglaan 100, PO Box 85500, 3508 GA Utrecht, The Netherlands; 3 Department of Orthodontics, Sophia Children’s Hospital, Erasmus University Medical Center, Wytemaweg 80, 3015 CN Rotterdam, The Netherlands; 4 Department of Plastic, Reconstructive and Hand Surgery, VU University Medical Center Amsterdam, De Boelelaan 1117, Postbus 7057, 1007 MB Amsterdam, The Netherlands

**Keywords:** Robin sequence, Cleft palate, Hypodontia, Tooth agenesis, Etiology

## Abstract

**Objectives:**

Partial tooth agenesis is frequently observed in Robin sequence. Tooth anomalies are increasingly considered as an extended phenotype of the cleft palate population. The study objective was to compare the prevalence and patterns of tooth agenesis in a group of patients with non-syndromic Robin sequence (ns-RS) and a group with non-syndromic cleft palate (ns-CP).

**Materials and methods:**

The panoramic radiographs of 115 ns-RS and 191 ns-CP patients were assessed for agenesis of the permanent dentition (excluding third molars) and the patterns recorded using the Tooth Agenesis Code.

**Results:**

Partial tooth agenesis was observed in 47.8% of ns-RS and 29.8% of ns-CP patients with a greater prevalence in the mandibula than in the maxilla, particularly in ns-RS. The teeth most frequently absent in both groups were the mandibular second premolars and maxillary lateral incisors. Tooth agenesis was bilateral in two-thirds of affected ns-RS patients and one-half of ns-CP patients. In ns-RS, bilateral agenesis of the mandibular second premolars was more frequently observed in female than that in male patients. Completely symmetrical patterns of hypodontia were found in around 45% of ns-RS patients with tooth agenesis compared to 35% in ns-CP. No association was found between the extent of the palatal cleft and the severity of hypodontia.

**Conclusion:**

Tooth agenesis is more prevalent in ns-RS than that in ns-CP, demonstrates a much greater predilection for the mandible in ns-RS, and bears no relation to the extent of the palatal cleft.

**Clinical relevance:**

When compared to ns-CP, additional developmental disturbances are likely involved in the etiology of tooth agenesis in ns-RS. Future research could help identify the underlying genetic traits and aid in classifying patients in those with and without expected tooth agenesis in order to facilitate orthodontic management strategies.

## Introduction

Although tooth agenesis is the most common developmental anomaly of the human permanent dentition, its etiology still remains poorly understood. Dental agenesis may occur either as an isolated trait or as part of a recognized congenital syndrome. Partial tooth agenesis or hypodontia is frequently observed in Robin sequence (RS). This congenital disorder bears the name of the French stomatologist Pierre Robin and consists of the triad of micro- or retrognathia, glossoptosis, and obstructive respiratory distress [1, 2]. In addition, the large majority of patients with RS are affected by a palatal cleft, though this is not universally perceived as an obligatory feature [3]. RS has been associated with a range of syndromes and chromosomal anomalies [4], yet is also encountered in isolation.

In the general, population tooth agenesis (excluding third molars) is observed in 3.2 to 7.6% of all individuals [5]. Recently, Norwegian [6] and Canadian [7] patient populations with isolated RS (ns-RS) were surveyed for the presence of hypodontia (excluding third molars), and a markedly increased prevalence was found in Norway with 42.3% as well as in Canada with 32.9%. In both of these studies, the dominant pattern of tooth agenesis was the bilateral absence of the mandibular second premolars. Moreover, Andersson et al. [6] demonstrated a positive correlation between the extent of the palatal cleft and the severity of hypodontia in their cohort of Norwegian patients. These findings lend credibility to the hypothesis that both cleft and tooth agenesis are manifestations of the same underlying tissue deficiency [8] with a possible shared genetic background [7, 9]. In the cleft palate patient population, tooth anomalies may therefore be considered as an extended phenotype [10]. Accordingly, if tooth agenesis in ns-RS is predominantly related to the same developmental disturbances underlying palatal clefting, similar prevalences of hypodontia and resembling patterns of tooth agenesis will be found in patients with ns-RS and in patients with an isolated cleft palate (ns-CP).

The objective of the present study was to test the aforementioned hypothesis indirectly by determining the prevalence of tooth agenesis (excluding third molars) and its patterns in a sample of Dutch patients with ns-RS and in a comparison group of Dutch patients with ns-CP. In order to record the pattern of tooth agenesis per individual patient, the study employed the Tooth Agenesis Code (TAC) which is a method that attaches a unique value to each pattern of agenesis [11, 12]. As a second objective, this study examined the relation between tooth agenesis and the extent of the palatal cleft in both groups of patients.

## Material and methods

In the Netherlands, all children born with an orofacial cleft are referred for evaluation to one of the 15 regional cleft teams, which offer multidisciplinary treatment according to general protocols. After a patient’s first consultation with a cleft treatment team, basic demographical data and characteristics of the orofacial malformations observed are registered with the overarching Dutch Association for Cleft Palate and Craniofacial Anomalies (*NVSCA*) in order to facilitate epidemiological and clinical research [13]. Four regional cleft treatment teams participated in this study: Alkmaar Medical Center, Erasmus Medical Center Rotterdam, University Medical Center Utrecht, VU Medical Center Amsterdam, and the Academic Center for Dentistry Amsterdam. Prior approval for this retrospective study was obtained from the Institutional Review Board of the Erasmus Medical Center Rotterdam (MEC 2014-183) and the University Medical Center Utrecht (METC 13/407). Dutch law did not require parental informed consent, since patients were not subject to investigational actions.

For the purpose of comparison, two distinct groups of patients were formed: (a) a study group of patients with ns-RS and (b) a comparison group of patients with ns-CP. Following the proposal by Breugem and Courtmanche [3], RS was defined as micrognathia, glossoptosis, and a history of obstructive respiratory distress. A second criterion for inclusion in the study group of patients was the manifestation of RS in isolation of other congenital malformations or syndromes. A retrospective review of the medical charts and, if available, the documentation of the relevant medical geneticist was conducted to verify whether all patients listed as RS in the internal institutional registries matched these criteria. Patients were selected for inclusion when one or more panoramic radiographs were available taken at age 7 years or later, as the evaluation of possible tooth agenesis of the permanent dentition (excluding third molars) is only possible from that age onwards [14]. A comparison group of consecutive patients with ns-CP was established through a similar procedure. The types and extent of the palatal clefts in both groups were obtained from the medical records. Seven categories of cleft palate were distinguished: (1) submucosal cleft, (2) incomplete cleft of the soft palate, (3) complete cleft of the soft palate, (4) complete cleft of the soft palate and submucosal cleft of the hard palate, (5) complete cleft of the soft palate and incomplete cleft of the hard palate, (6) complete cleft up to incisive foramen, and (7) unknown (cleft palate present but type unknown).

The panoramic radiographs of all patients were screened twice for the presence of tooth agenesis (excluding third molars) by a single researcher (AdS) with an interval of 2 weeks. A tooth was deemed to be congenitally absent when no mineralization of its crown was visible. Subsequently, the exact patterns of tooth agenesis were recorded in File Maker Pro 12.0 (Filemaker Inc., Santa Clara, CA, USA) using the TAC [Van Wijk, Créton] [11, 12], which is a binary system that attaches a unique value to each pattern of tooth agenesis per dental quadrant. Detailed information on the TAC is available at http://www.toothagenesiscode.com/. Statistical analysis was performed with IBM SPSS Statistics 21.0 (IBM Inc., New York, NY, USA) using the Chi-square test and one-way ANOVA. A *P* value of less than 0.05 was considered statistically significant. Intraobserver agreement between the first and second screening of the panoramic radiographs was evaluated using Cohen’s kappa and was considered excellent (kappa of 1.0). Interobserver agreement was assessed by letting a second researcher (RH) screen the radiographs of a random subset of 15 patients and compare these to the ratings of the first researcher (AdS). This produced a Cohen’s kappa of 0.91 indicating a high degree of consensus.

## Results

### Prevalence of tooth agenesis

A total of 115 patients with ns-RS were included in the study group and 191 patients with ns-CP in the comparison group, see Table 1. The panoramic radiographs used for analysis were taken during 2001–2014 for the study group and during 1991–2007 for the comparison group.

**Table 1:** Prevalence of tooth agenesis

	Non-syndromic Robin sequence (ns-RS)		Non-syndromic cleft palate (ns-CP)		Chi-square Test (two-sided)
	*N*	%	*N*	%	*P*
Total number of patients	115	100	191	100	
Males/females	49/66	42.6/57.4	86/105	45.0/55.0	
Tooth agenesis (excl. 3rd molars)	55	47.8	57	29.8	**< 0.001**
Maxilla	28	24.3	31	16.2	**0.082**
Mandibula^a^	45	39.1	39	20.4	**< 0.001**
Right dental quadrants (q1, q4)	42	36.5	43	22.5	**0.008**
Left dental quadrants (q2, q3)	50	43.4	42	22.0	**< 0.001**
In males	18/49	36.7	28/86	32.6	0.622
In females^b^	37/66	56.0	30/105	28.6	**< 0.001**

^a^Compared to maxillary tooth agenesis (Chi-square test—two-sided): RS *P* = **0.016**; CP *P* = 0.290

^b^Compared to tooth agenesis in males (Chi-square test—two-sided): RS *P* = **0.040**; CP *P* = 0.551

Table 1 shows that hypodontia (excluding third molars) was observed in 47.8% of patients with ns-RS compared to a significantly lower 29.8% of patients with ns-CP. In both groups, mandibular tooth agenesis was observed more frequently than maxillary tooth agenesis, though only in ns-RS, this difference was significant. The prevalence of hypodontia in the right dental quadrants did not differ significantly from that in the left quadrants in either group. Tooth agenesis in ns-RS was significantly more prevalent in females than that in males. In ns-CP, no significant difference was found in the prevalence of hypodontia between males and females.

In ns-RS, a total of 139 teeth were absent with a median of 2 per patient and a range of one to ten teeth per patient. Table 2 illustrates that 64.0% of missing teeth were second premolars and 18.7% lateral incisors. In the comparison group of ns-CP, a number of 106 teeth were missing with a median of 2 per patient ranging from one to five. Again, a majority of 67.6% were second premolars and 22.6% were lateral incisors.

**Table 2:** Prevalence of tooth agenesis per tooth type

A: Non-syndromic Robin sequence (ns-RS)						
Tooth number	Maxilla right	Maxilla left	Mandibula left	Mandibula right	All dental quadrants	
	(q1, *N*)	(q2, *N*)	(q3, *N*)	(q4, *N*)	*N*	%
1	0	0	1	2	3	2.2
2	6	9	8	3	26	18.7
3	0	0	0	1	1	0.7
4	4	3	4	4	15	10.8
5	12	17	31	29	89	64.0
6	1	0	0	0	1	0.7
7	1	1	2	0	4	2.9
Total	24	30	46	39	139	100
B: Non-syndromic cleft palate (ns-CP)						
1	0	0	0	3	3	2.8
2	10	11	2	1	24	22.6
3	0	0	0	0	0	0.0
4	1	1	2	1	5	4.7
5	12	11	25	24	72	67.6
6	0	0	0	0	0	0.0
7	0	1	0	1	2	1.9
Total	23	24	29	30	106	100

### Patterns of tooth agenesis

Tables 3 and 4 show that in the study group of ns-RS patients, 28 different patterns of tooth agenesis were seen in the panoramic radiographs. In non-syndromic RS, the dominant patterns of tooth agenesis were the bilateral absence of the mandibular second premolars (TAC 0.0.16.16) in 9.6% of patients, the absence of the left mandibular second premolar (TAC 0.0.16.0) in 5.2%, and the absence of all second premolars (TAC 16.16.16.16) in 4.3%. One or more mandibular second premolars were absent in 15 of 28 patterns. Bilateral absence of the mandibular second premolars was observed in 20.0% (*N* = 11/55) of ns-RS patients with tooth agenesis and was observed more frequently in female patients (Chi-square test, two-sided, *P* = 0.015). The most prevalent patterns with agenesis of the lateral incisors were bilateral mandibular/maxillary agenesis (TAC 0.0.2.2/2.2.0.0) in 4.3% of ns-RS patients and left unilateral agenesis (TAC 0.2.0.0) in 1.7%. The maxillary lateral incisors were absent in 7 of 28 patterns and the mandibular lateral incisors in 5 of 28 patterns.

**Table 3:** Patterns of tooth agenesis based on TAC per dental quadrant

A. Non-syndromic Robin sequence (ns-RS)		q1		q2		q3		q4	
TAC	Tooth type^a^	*N*	%	*N*	%	*N*	%	*N*	%
0	none	94	81.7	88	76.5	76	66.1	81	70.4
1	I_1_	0	0.0	0	0.0	0	0.0	2	1.7
2	I_2_	6	5.2	8	7.0	6	5.2	3	2.6
8	P_1_	2	1.7	2	1.7	1	0.9	0	0.0
16	P_2_	10	8.7	14	12.2	28	24.3	24	20.9
18	P_2_ + I_2_	0	0.0	1	0.9	0	0.0	0	0.0
20	P_2_ + C	0	0.0	0	0.0	0	0.0	1	0.9
24	P_2_ + P_1_	1	0.9	1	0.9	1	0.9	4	3.5
26	P_2_ + P_1_ + I_2_	0	0.0	0	0.0	1	0.9	0	0.0
32	M_1_	1	0.9	0	0.0	0	0.0	0	0.0
67	I_1_ + I_2_ + M_2_	0	0.0	0	0.0	1	0.9	0	0.0
80	P_2_ + M_2_	0	0.0	1	0.9	0	0.0	0	0.0
88	P_2_ + P_1_ + M_2_	1	0.9	0	0.0	1	0.9	0	0.0
Total		115	100	115	100	115	100	115	100
No. of TAC patterns		7		7		8		6	
B. Non-syndromic cleft palate (ns-CP)		q1		q2		q3		q4	
TAC	Tooth type	*N*	%	*N*	%	*N*	%	*N*	%
0	none	170	89.0	169	88.5	162	84.8	161	84.3
1	I_1_	0	0.0	0	0.0	0	0.0	3	1.6
2	I_2_	9	4.7	9	4.7	2	1.0	1	0.5
8	P_1_	0	0.0	1	0.5	2	1.0	1	0.5
16	P_2_	10	5.2	9	4.7	25	13.1	24	12.6
18	P_2_ + I_2_	1	0.5	2	1.0	0	0.0	0	0.0
24	P_2_ + P_1_	1	0.5	0	0.0	0	0.0	0	0.0
64	M_2_	0	0.0	1	0.5	0	0.0	1	0.5
Total		191	100	191	100	191	100	191	100
No. of TAC patterns		5		6		4		6	

*I*_*1*_ central incisor, *I*_*2*_ lateral incisor, *P*_*1*_ first premolar, *P*_*2*_ second premolar, *C* canine, *M*_*1*_ first molar, *M*_*2*_ second molar

**Table 4:** Patterns of tooth agenesis—TAC patterns

A. Non-syndromic Robin sequence (ns-RS)					
No.	TAC value	Frequency (*N*)	Percentage (%)	Missing teeth (*N*)	Missing tooth/teeth
1	0.0.0.0	60	52.2	0	none
2	0.0.16.16	11	9.6	2	35, 45
3	0.0.16.0	6	5.2	1	35
4	16.16.16.16	5	4.3	4	15, 25, 35, 45
5	16.16.0.0	3	2.6	2	15, 25
6	0.0.2.2	3	2.6	2	32, 42
7	0.0.0.16	3	2.6	1	45
8	0.2.0.0	2	1.7	1	22
9	0.16.0.0	2	1.7	1	25
10	2.2.0.0	2	1.7	2	12, 22
11	8.8.2.0	1	0.9	3	14, 24, 32
12	88.80.88.24	1	0.9	10	17, 15, 14, 25, 27, 37, 35, 34, 44, 45
13	2.2.16.20	1	0.9	5	12, 22, 35, 43, 45
14	0.0.67.1	1	0.9	4	37, 32, 31, 41
15	16.16.2.0	1	0.9	3	15, 25, 32
16	2.2.0.16	1	0.9	3	12, 22, 45
17	0.8.8.0	1	0.9	2	24, 34
18	16.16.26.24	1	0.9	7	15, 25, 35, 34, 32, 44, 45
19	8.2.0.0	1	0.9	2	14, 22
20	2.2.16.16	1	0.9	3	12, 22, 35, 45
21	24.24.16.24	1	0.9	7	15, 14, 24, 25, 35, 44, 45
22	0.0.0.1	1	0.9	1	41
23	0.0.2.0	1	0.9	1	32
24	32.0.0.16	1	0.9	2	17, 45
25	0.16.16.6	1	0.9	3	25, 35, 45
26	2.18.16.16	1	0.9	5	12, 22, 25, 35, 45
27	0.16.16.0	1	0.9	2	25, 35
28	0.0.24.24	1	0.9	4	35, 34, 44, 45
	Total	115	100		
B. Non-syndromic cleft palate (ns-CP)					
No.	TAC value	Frequency (*N*)	Percentage (%)	Missing teeth (N)	Missing tooth/teeth
1	0.0.0.0	134	70.2	0	None
2	0.0.16.16	10	5.2	2	35, 45
3	0.0.0.16	6	3.1	1	35
4	0.0.16.0	5	2.6	1	35
5	2.2.0.0	5	2.6	2	12, 22
6	2.0.0.0	3	1.6	1	12
7	16.0.16.16	3	1.6	3	15, 35, 45
8	0.2.0.0	3	1.6	1	22
9	16.16.16.16	3	1.6	4	15, 25, 35, 45
10	16.0.0.0	2	1.0	1	15
11	16.16.0.0	2	1.0	2	15, 25
12	0.0.2.0	2	1.0	1	32
13	18.18.16.0	1	0.5	5	15, 12, 22, 25, 35
14	0.8.8.8	1	0.5	3	24, 34, 44
15	0.18.16.16	1	0.5	4	22, 25, 35, 45
16	0.64.0.0	1	0.5	1	27
17	0.0.0.1	1	0.5	1	41
18	0.16.16.16	1	0.5	3	25, 35, 45
19	0.0.0.2	1	0.5	1	42
20	0.0.0.64	1	0.5	1	47
21	0.16.16.0	1	0.5	2	25, 35
22	2.2.8.1	1	0.5	4	12, 22, 34, 41
23	0.16.0.0	1	0.5	1	25
24	0.16.0.1	1	0.5	2	25, 41
25	24.0.0.0	1	0.5	2	15, 14
	Total	191	100		

Bilateral tooth agenesis was observed in 17 patterns or 65.6% (*N* = 36/55) of ns-RS patients with tooth agenesis, while unilateral agenesis was seen in 11 patterns or 34.5% (*N* = 19/55) of patients. A completely symmetrical pattern of tooth agenesis was noted in 6 patterns or 45.5% (*N* = 25/55) of patients with hypodontia. In maxillary agenesis, 60.7% (*N* = 17/28) of patients had a symmetrical pattern compared to 48.9% (*N* = 22/45) with mandibular tooth agenesis.

**Fig. 1:**
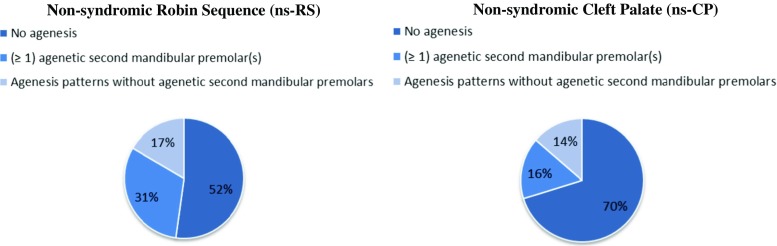
Prevalence of agenesis of the mandibular second premolars

As presented in Tables 3 and 4, the comparison group of ns-CP patients had 25 different patterns of tooth agenesis in the complete dentition. In close resemblance to the findings in ns-RS, the dominant patterns of tooth agenesis were bilateral absence of the mandibular second premolars (TAC 0.0.16.16) in 5.2% of all ns-CP patients and unilateral mandibular absence of the mandibular second premolars (TAC 0.0.016 and 0.0.16.0) in 5.7%. Bilateral absence of the mandibular second premolars was seen in 17.5% (*N* = 10/57) of ns-CP patients with dental agenesis, which did not differ significantly from the situation in ns-RS as shown in Table 5. Patients with ns-RS were significantly more likely to have one or more agenetic second premolars compared to patients with ns-CP (Chi-square test, two-sided, *P* = 0.002). Figure 1 provides a graphic interpretation of this difference. Absence of the lateral incisors in ns-CP was most frequently observed in the maxilla, either bilaterally (TAC 2.2.0.0) in 2.6% of all ns-CP patients or unilaterally (TAC 2.0.0.0) in 1.6%.

**Table 5:** Symmetry in patients with dental agenesis

	Non-syndromic Robin sequence (ns-RS)		Non-syndromic cleft palate (ns-CP)		Chi-square test (two-sided)
Pattern	*N*	*%*	*N*	*%*	*P*
Bilateral agenesis	36/55	65.4%	30/57	52.6%	0.168
Of mandibular 2nd premolars	11/55	20.0%	10/57	17.5%	0.739
Of maxillary lateral incisors	4/55	7.3%	6/57	10.5%	0.546
Complete symmetry	25/55	45.5%	20/57	35.1%	0.263
Maxillary symmetry	17/28	60.7%	12/31	38.7%	0.091
Mandibular symmetry	22/45	48.9%	19/39	48.7%	0.988

In ns-CP, 12 patterns included bilateral agenesis translating into 52.6% (*N* = 30/57) of patients with tooth agenesis. Symmetrical patterns of tooth agenesis were seen in 4 patterns or 35.1% (*N* = 20/57) of patients with hypodontia. In patients with maxillary agenesis, 38.7% (*N* = 12/31) had a symmetrical pattern against 48.7% (*N* = 19/39) of patients with mandibular agenesis. Table 5 demonstrates that formal testing did not reveal a significant difference between ns-CP and ns-RS in the prevalences of symmetrical patterns of dental agenesis.

### Tooth agenesis and cleft extent

Table 6 shows the distribution of palatal cleft types in the study group of ns-RS patients and in the comparison group of ns-CP patients. The relation between the palatal cleft extent and the mean number of missing teeth was analyzed using one-way ANOVA, excluding patients without tooth agenesis and all cleft palate types with fewer than 10 patients. In both ns-RS (*P* = 0.539) and ns-CP (*P* = 0.947), there was no significant association between the extent of the palatal cleft and tooth agenesis.

**Table 6:** Distribution of types of cleft palate

No.	Type of cleft palate	Non-syndromic Robin sequence (ns-RS)	Non-syndromic cleft palate (ns-CP)
1	Soft palate: submucosal	1	29
2	Soft palate: incomplete	2	8
3	Soft palate: complete	13	42
4	Soft palate: complete; hard palate: submucosal	0	2
5	Soft palate: complete; hard palate: incomplete	41	41
6	Complete cleft up to incisive foramen	26	49
7	Unknown (cleft palate present, type unknown)	32	20

## Discussion

The objectives of this study were to determine the prevalence of tooth agenesis (excluding third molars) and its patterns in a Dutch population of patients with ns-RS and ns-CP and to test the hypothesis that tooth agenesis in ns-RS is mainly related to the developmental disturbances that also produced the palatal cleft seen in the majority of patients. The present study included 115 patients with ns-RS and observed tooth agenesis in 47.8% of patients. This number corresponds roughly to the prevalence rates for tooth agenesis in ns-RS previously found in Canada [7] (32.9%) and in Norway [6] (42.3%), as well as to the rates observed for patient samples including syndromic cases in Sweden [15] (35.7%) and Finland (50%) [16]. Contrary to the aforementioned Canadian and Norwegian studies, we found a significantly greater prevalence of hypodontia in female patients. In the Swedish study by Larsson et al. [15] and the Finnish study by Rintala et al. [16], no distinction was made between male and female patients. This finding matches the result of a large meta-analysis of tooth agenesis in Caucasian populations, which showed that females are 1.37 times more susceptible to tooth agenesis than males [5]. The influence of sex in the pathophysiology of tooth agenesis remains unclear though.

In the comparison group of 191 patients with ns-CP, the prevalence of tooth agenesis was lower with 29.8%, which lies within the range of 21.0–36.8% described for ns-CP in other patient populations [17–19]. Again, tooth agenesis demonstrated a (non-significant) tendency to a greater prevalence in the mandibula than the maxilla, matching the findings of Aizenbud et al. [20] in an Israeli patient population. However, a maxillary predominance in ns-CP was observed in Finland by Ranta and Tulensalo [21] and in New York by Shapira et al. [22], though the latter study only included 9 patients with hypodontia. In both ns-CP and ns-RS, the most commonly missing teeth were the second premolars in both dental arches and the maxillary lateral incisors, which matches the findings of earlier studies [7, 20–23]. Although the second premolars and lateral incisors are also the most frequently absent teeth after the third molars in the general population [5], the prevalence of tooth agenesis is substantially higher in the patient groups studied.

With regards to the patterns of hypodontia, this study showed that in line with the literature [6, 7, 20], tooth agenesis occurred more often in the lower dental arch than in the upper arch. Furthermore, in ns-RS, tooth agenesis was shown to present itself bilaterally in two-thirds of patients, analogous to the findings of Andersson et al. [6] for Norway and Antonarakis and Suri [7] for Canada. As in the latter study, a completely symmetrical pattern was seen in around 45% of ns-RS patients with tooth agenesis. Moreover, similar to the Norwegian and Canadian studies, the teeth most frequently absent were the second premolars in both arches followed by the maxillary lateral incisors. Although mandibular tooth agenesis was observed more frequently in ns-RS, the occurrence of bilateral and symmetrical patterns of agenesis was not significantly different in ns-CP. Interestingly, nearly all patterns of tooth agenesis per quadrant observed in ns-CP were also seen in ns-RS, though in the latter group, additional patterns were recorded.

This study also examined the relationship between the extent of the palatal cleft and the degree of hypodontia. Developmental anomalies of the dentition are increasingly considered to be a subphenotype of the cleft population since both teeth and lip/palate are derived from the branchial arch precursors and influenced by related morphogenetic patterning signals [4]. Indeed, several candidate genes have been associated with both dental anomalies and clefting: *IRF6*, *MSX1*, *PAX9*, and *TGFB3* [4, 9]. Moreover, a small number of earlier studies found a positive correlation (of unknown strength) between the cleft extent and the severity of dental agenesis in patients with ns-RS [6] and ns-CP [21]. However, the present study found no evidence for any association in either group of patients.

In summary, this study showed a greater prevalence of tooth agenesis in ns-RS relative to ns-CP, a more pronounced mandibular agenesis in ns-RS, and the absence of a direct relationship between the extent of the palatal cleft and hypodontia in ns-RS. Consequently, additional (or even different) developmental disturbances are probably involved in tooth agenesis in ns-RS, with disturbances related to mandibular hypoplasia, the most likely candidates. The failure of mandibular outgrowth in ns-RS is hypothesized to result from intrauterine constraints on the mandible [24], defects in both the generation and growth of Meckel’s cartilage (the first mandibular skeletal element) [25], as well muscular defects with failure of tongue descent [4, 26, 27]. Mandibular hypoplasia could lead to hypodontia through spatial constraints with agenesis of the last of a class of teeth to develop, such as the mandibular second premolars. The prevalence of agenesis of the maxillary over the mandibular lateral incisors could similarly be explained by spatial constraints caused by the development of the canine and the later calcification of the maxillary lateral incisors [28]. Alternatively, the developmental vulnerability of the maxillary lateral incisor may be attributed to the complex origin of its germ at the site of fusion between the medial nasal and maxillary facial outgrowths [29, 30]. However, mandibular hypodontia in ns-RS could also have a more direct etiology in defects in the genes regulating odontogenesis, particularly in patients with agenesis of deciduous precursors. Unfortunately, the genetic background of RS remains poorly understood with only mutations in the *SOX9* gene implicated in the non-syndromic form of the condition [4]. Subphenotypes of ns-RS with mandibular hypodontia have diminished mandibular dimensions and a different facial morphology compared to those without hypodontia [31, 32]. Therefore, future research efforts into the genetic traits of these patient populations could allow for a more tailored planning of orthodontic treatment and/or orthognathic surgery.

The strength of this study is the inclusion of a comparison group of patients with ns-CP and the use of the TAC system which precisely elucidated the differences in the prevalences and patterns of tooth agenesis between ns-RS and ns-CP. In addition, this study examined the relationship between the degree of hypodontia and the extent of the palatal cleft using formal statistical analysis. Despite these qualities, the study is subject to several limitations. First, it has a retrospective character without a strict case-control design. Second, the study did not assess tooth agenesis in the deciduous dentition.

## Conclusions

Tooth agenesis is more prevalent in ns-RS than that in ns-CP, demonstrates a much greater predilection for the mandible in ns-RS, and bears no relation to the palatal cleft. These findings suggest that additional developmental disturbances are likely involved in the etiology of tooth agenesis in ns-RS when compared to ns-CP. Future research could help identify the underlying genetic traits and aid in classifying patients in those with and without expected tooth agenesis in order to facilitate orthodontic management strategies.
